# Three-year assessment of integrated vector management in Attica, Greece: results from surveillance and control activities

**DOI:** 10.1038/s42003-025-08825-y

**Published:** 2025-11-06

**Authors:** Pascale C. Stiles, Georgios Balatsos, Prasad Liyanage, Jerome N. Baron, Maria Sakellariou Sofianou, Marina Bisia, Vasileios Karras, Till Bärnighausen, Eleni Patsoula, Joacim Rocklöv, Antonios Michaelakis, Aditi Bunker

**Affiliations:** 1 Heidelberg Institute of Global Health (HIGH), Heidelberg University, Heidelberg, Germany; 2 Laboratory of Insects & Parasites of Medical Importance, Benaki Phytopathological Institute, Kifisia, Greece; 3 Unit of Medical Entomology, Laboratory for the Surveillance of Infectious Diseases (LSID), Division of Infectious, Parasitic Diseases and Zoonoses, Department of Public Health Policy, School of Public Health, University of West Attica, Athens, Greece; 4 Department of Global Health and Population, Harvard T.H. Chan School of Public Health, Boston, MA USA; 5 Africa Health Research Institute, Durban, South Africa; 6 Interdisciplinary Centre for Scientific Computing, Heidelberg University, Heidelberg, Germany; 7 Department of Epidemiology and Global Health, Umeå University, Umeå, Sweden

**Keywords:** Entomology, Infectious diseases

## Abstract

The West Nile virus vector *Culex pipiens* and invasive species *Aedes albopictus* are abundant in Attica, Greece, which deploys an integrated vector management program for targeted vector control. The objective of our study is to evaluate the effects of this program on mosquito vector populations. Using mosquito surveillance and intervention occurrence data, we assessed the effects of mosquito control interventions on vector populations using a two-stage interrupted time series (ITS) approach. First, we fitted ITS models to 16 weeks of data centered on the week of each unique species-specific intervention. Second, we pooled the estimated coefficients in a meta-regression model. Following vector control interventions at the targeted intervention sites, we observed an overall 34% reduction (RR 0.66; 95% CI 0.50-0.89) in *Culex pipiens* counts and a nonsignificant 5% increase (RR 1.05; 95% CI 0.81-1.35) in *Aedes albopictus* counts, compared to the pre-intervention period. These results support the implementation in reducing *Culex pipiens* populations in Attica as part of an integrated public health plan to mitigate West Nile virus risk. However, the limited impact on *Ae. albopictus* suggests the need for complementary strategies beyond conventional biocides. Overall, this study could serve a model for evaluating IVM programs in diverse settings.

## Abstract (web summary)

Interrupted time series models evaluate the impact of a mosquito control program, providing support for the continued implementation of the program against *Cx. pipiens* but suggesting the need for complementary strategies against *Ae. Albopictus*.

## Introduction

Climate change is leading to an expansion both in geographical range and seasonality of vector-borne disease (VBD) transmission in Europe^1^. The *Culex* (*Cx*.) *pipiens* complex is a group of native European mosquito species capable of transmitting West Nile virus (WNV)^2,3^. West Nile virus was first recorded in Uganda in 1937 and has spread quickly to become endemic in parts of Europe and North America^4^. In particular, the eastern Mediterranean region experiences some of the highest WNV incidences in the European Union^5,6^. Transmission of WNV has been an annual public health concern in Greece since 2010, when Greece experienced a major WNV outbreak. Recently, in 2022, a large WNV outbreak in Greece resulted in 286 diagnosed cases and 33 deaths^7–10^. Rising ambient temperatures related to climate change are expected to cause a longer duration of WNV transmission season as well as an expansion of areas at risk for WNV due to increased vectorial capacity in *Cx. pipiens* populations^1,11,12^. The overwintering of WNV in active mosquito vectors captured in adult mosquito traps was detected for the first time in Greece in 2022^13^. In contrast to other studies worldwide, which focused on the detection of WNV in hibernating mosquitoes^14–16^, this highlights the continued outdoor activity of *Cx. pipiens* and the possible circulation of WNV throughout the winter months^13^.

Furthermore, the invasive species *Aedes albopictus*, commonly known as the tiger mosquito, has expanded its presence in Europe in recent years, due to increased trade and travel and climate change^17,18^. Its competence as a mosquito vector for dengue, chikungunya, and Zika viruses is well-established^19,20^. This species has been responsible for autochthonous transmission of these arboviruses in France, Italy, and Spain, including a small dengue cluster as far north as the Paris region and a wide-scale outbreak in Italy in 2024^21–25^. The *Ae*. *albopictus* species was first detected in Corfu and Thesprotia, Greece, in 2003 and has since become established throughout the country^26^. Although no autochthonous transmission related to this invasive species has yet been recorded, exotic arbovirus infections are regularly imported into Greece by travelers^27,28^. Warmer winter temperatures, driven by climate change, have also led to year-long detection of *Ae. albopictus* adults in Athens, lengthening the potential transmission season^29^.

With no approved human vaccine for WNV and only recent authorization in Europe of vaccines against dengue and chikungunya viruses^30–32^, mosquito vector control and practice of personal prevention methods to limit exposure remain the primary means for disease prevention. Mosquito vector control programs aim to reduce immature and/or adult mosquito populations quickly and effectively. These programs are typically applied during the seasons in which mosquitoes are active and are further enhanced in periods of high epidemic risk, such as when locally acquired human WNV infections or other mosquito-borne arboviral cases are recorded or when viral circulation in mosquitoes is confirmed^13,33–36^. The design, surveillance, and implementation of integrated vector management (IVM) programs are public (and veterinary) health interventions aiming to protect human and animal populations and/or address outbreaks of VBDs, such as WNV. Integrated vector management programs, however, require annual statewide investment and coordination across public health stakeholders, state officials, and/or private agencies. Such programs are critical to inform resource allocation to targeted areas with the highest risk of VBD transmission and can be adapted to various vector species and pathogens^37^, but are resource-intensive. Furthermore, the direct effects of control actions on mosquito vector populations are often difficult to estimate as well as communicate to stakeholders, who may be reluctant to make such financial investments^34,38^. Since 2021, the Benaki Phytopathological Institution (BPI) and the Public Health Agency of Attica Region have expanded traditional mosquito vector control activities into an IVM program at a cost of 364,500 Euros for the period 2021–2023. The program included a year-round mosquito vector surveillance system with coverage across the entire region and was successful in collecting critical information on their composition and numbers (trap counts) to guide appropriate control and strategic decision-making. As part of this system, weekly trap collections and morphological mosquito identifications were conducted, alongside the creation of interactive maps, which were updated weekly. These maps provided public health officials responsible for mosquito control in the Attica Region (local authorities) with an overview of mosquito population trends in the area, enabling them to monitor increases, evaluate the effectiveness of mosquito vector control interventions, and identify potential hotspots. In case the problem persisted, local authorities conducted site visits to identify breeding sites that may be contributing to the problem, so that they can implement targeted and effective interventions.

The higher numbers of *Ae. albopictus* and *Cx. pipiens*, as well as the detection of WNV-positive *Cx. pipiens*, in the Attica Region^36,39^, provide an opportunity to evaluate mosquito vector control implementation where capacity building and evidence-based interventions are critical. There is also an important historical experience in managing VBDs, as Greece’s successful elimination of malaria in 1974 through large-scale mosquito vector control programs demonstrates the country’s capacity to implement effective mosquito management when supported by sustained intervention strategies and surveillance systems^40,41^.

To our knowledge, there is a gap in the literature concerning decision-making protocols for implementing IVM. While existing research provides evidence-based guidance on IVM strategies, operational protocols for implementation remain highly context-dependent and are rarely documented in a generalizable format. While surveillance data are often used to guide mosquito vector control efforts, standardized criteria within a generic decision tool are missing for initiating specific actions, such as biocide application or community engagement campaigns. In our current study, we aim to support the development of decision-making protocols in collaboration with regional stakeholders for initiating interventions within the IVM program by highlighting the activities of the IVM program and evaluating the effects of the IVM program on mosquito vector populations in the Attica Region, Greece. Our findings may contribute to improving IVM and strategic decision-making not only in the Attica Region but also in other countries with similar mosquito vector challenges. By establishing a replicable framework for evaluating mosquito vector control programs, our objective is to ensure that future interventions are both timely and strategically targeted.

## Results

### Entomological surveillance

**Fig. 1:**
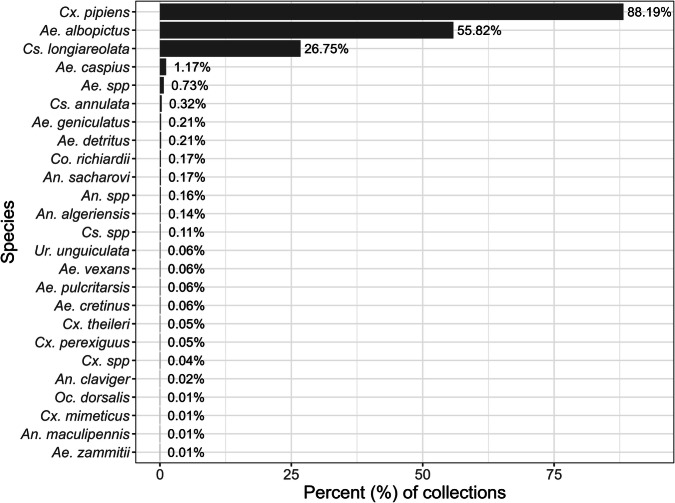
Proportional presence of mosquito species. Proportion of 8044 valid sampling events in which a given species was detected. Sampling events occurred from January 2021 through December 2023, and species detection was not mutually exclusive; thus, proportions do not sum to 100%.

**Fig. 2:**
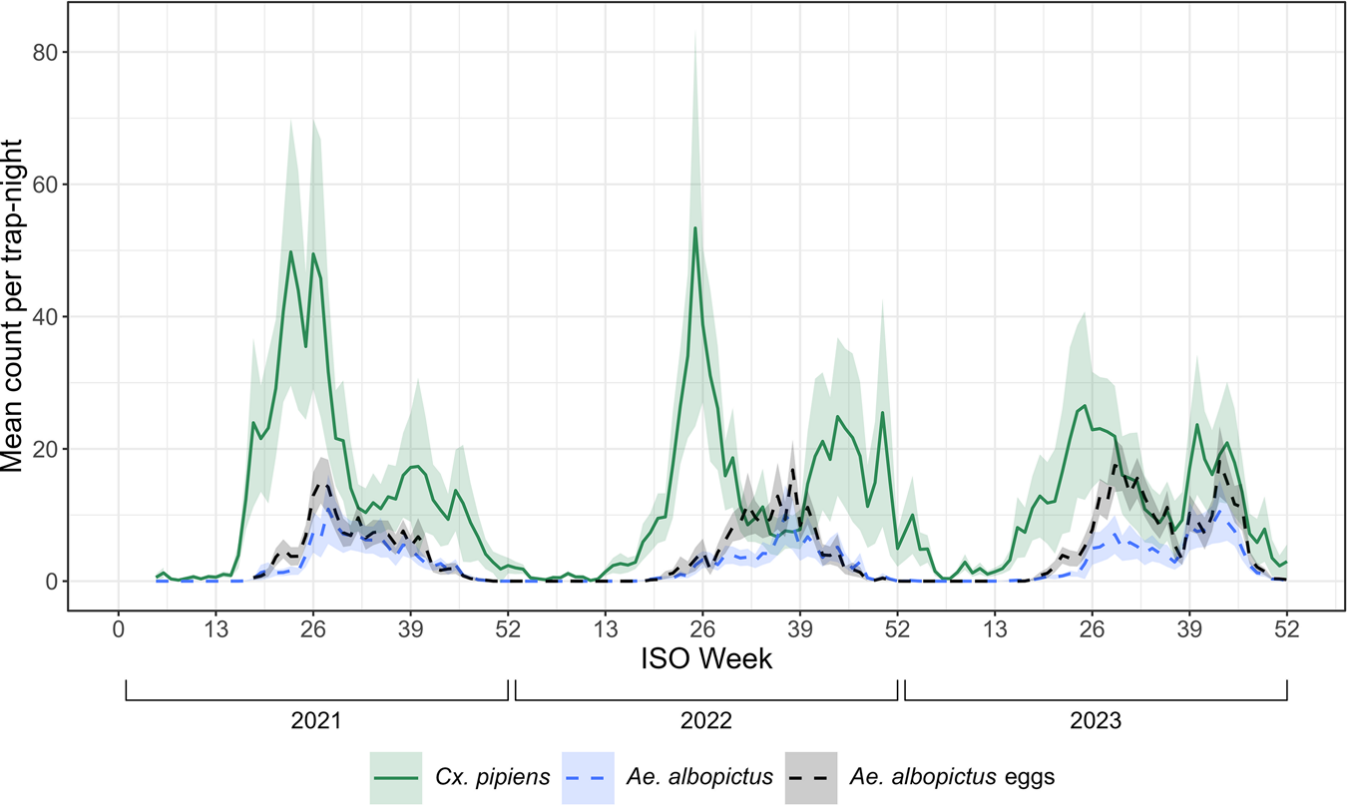
Mean weekly mosquito count per trap-night. The mean weekly mosquito count per trap-night over three years (January 2021 to December 2023, 52 weeks per year). The solid green line represents *Cx. pipiens* adults, and a broken blue line represents *Ae. albopictus* adults, both recorded in BG-Sentinel traps. The broken black line represents *Ae. albopictus* eggs, recorded in ovitraps. The 95% confidence intervals are represented in the shaded area during each week of the study period. This is produced from all 8044 valid sampling events over 156 weeks.

Between January 2021 and December 2023, there were 8408 collections across the 57 trap locations. We excluded 364 (4.3%) of these collections due to unreliable counts. The most common reason for exclusion was the presence of ants or other predatory insects in the trap (186/364). *Culex pipiens* was the most frequently collected mosquito species, being present in 7,094 (88.2%) of the collections, followed by *Ae. albopictus* in 4490 (55.8%) and *Culiseta longiareolata* in 2152 (26.8%) collections. In addition, certain *Anopheles* species, which are competent vectors for malaria, were also recorded (Fig. 1). We collected *Cx. pipiens* specimens in at least one trap during every week of the study period and this species was generally observed in higher trap counts than *Ae. albopictus* (Fig. 2).

The trap counts of *Cx. pipiens* and *Ae. albopictus* exhibited distinct patterns over the study period (Fig. 2), which is expected given their differences in biology, including preferred breeding sites and responses to climatic conditions. Based on the results, *Cx. pipiens* is active throughout the year, while *Ae. albopictus* activity begins late spring (late-mid May), and additionally, we recorded that *Ae. albopictus* always peaks later than *Cx. pipiens*. More specifically, adult female *Cx. pipiens* traps counts peaked in weeks 23–26 (June) in 2021, 24–28 (mid-June through mid-July) in 2022, and 40–44 (October) in 2023. We observed a shorter period of depressed winter activity for *Cx. pipiens* during the winter months of 2022–2023 compared to the previous winter. In contrast, *Ae. albopictus* adult females reached peak trap counts later than *Cx. pipiens*, peaking in weeks 27–29 (early July) in 2021, 37–38 (mid-September) in 2022, and 39–45 (mid-September through late October) in 2023 (Fig. 2). Meanwhile, the first egg counts of *Ae. albopictus* were recorded in week 18 (late April) of 2021, week 19 (early May) in 2022, and week 16 (mid-April) in 2023, implying the beginning of its activity each year.

During the study period, 52 unique mosquito control interventions were enacted, 25 (48.1%) of which were in reaction to high *Cx. pipiens* trap counts, 12 (23.1%) in reaction to high *Ae. albopictus* trap counts, and 15 (28.8%) in reaction to high trap counts of both species. The public health officials who are responsible for mosquito control in the Attica Region primarily targeted *Cx. pipiens* mosquito larvae during interventions, with only 3 applications (12.0%) targeting the adult stage alone. In contrast, the larval stage was never the sole target for interventions against *Ae. albopictus* (Table 1, Supplementary Table 1) since their breeding sites can be difficult to find because they are often cryptic and located in private areas or urban environments.

**Table 1:** Summary of mosquito vector control interventions

		Species target				
		*Aedes albopictus*	*Culex pipiens*	Both species	Total	*P*-value^a^
		*n* (%)	*n* (%)	*n* (%)	*n* (%)	
Life stage target	Adults	5 (41.7%)	3 (12.0%)	8 (53.3%)	16 (30.8%)	
	Larvae	0 (0.0%)	11 (44.0%)	7 (46.7%)	18 (34.6%)	
	Both	7 (58.3%)	11 (44.0%)	0 (0.0%)	18 (34.6%)	
	Total	12 (100.0%)	25 (100.0%)	15 (100.0%)	52 (100.0%)	<0.001

Data were stratified by species and life stage target. Interventions were deployed in response to a high number of *Ae. albopictus* or *Cx. pipiens* counts, or both species in conjunction. Interventions were directed against adult mosquitoes, larvae, or both adult and larval stages together.

^a^Fisher’s exact test.

**Fig. 3:**
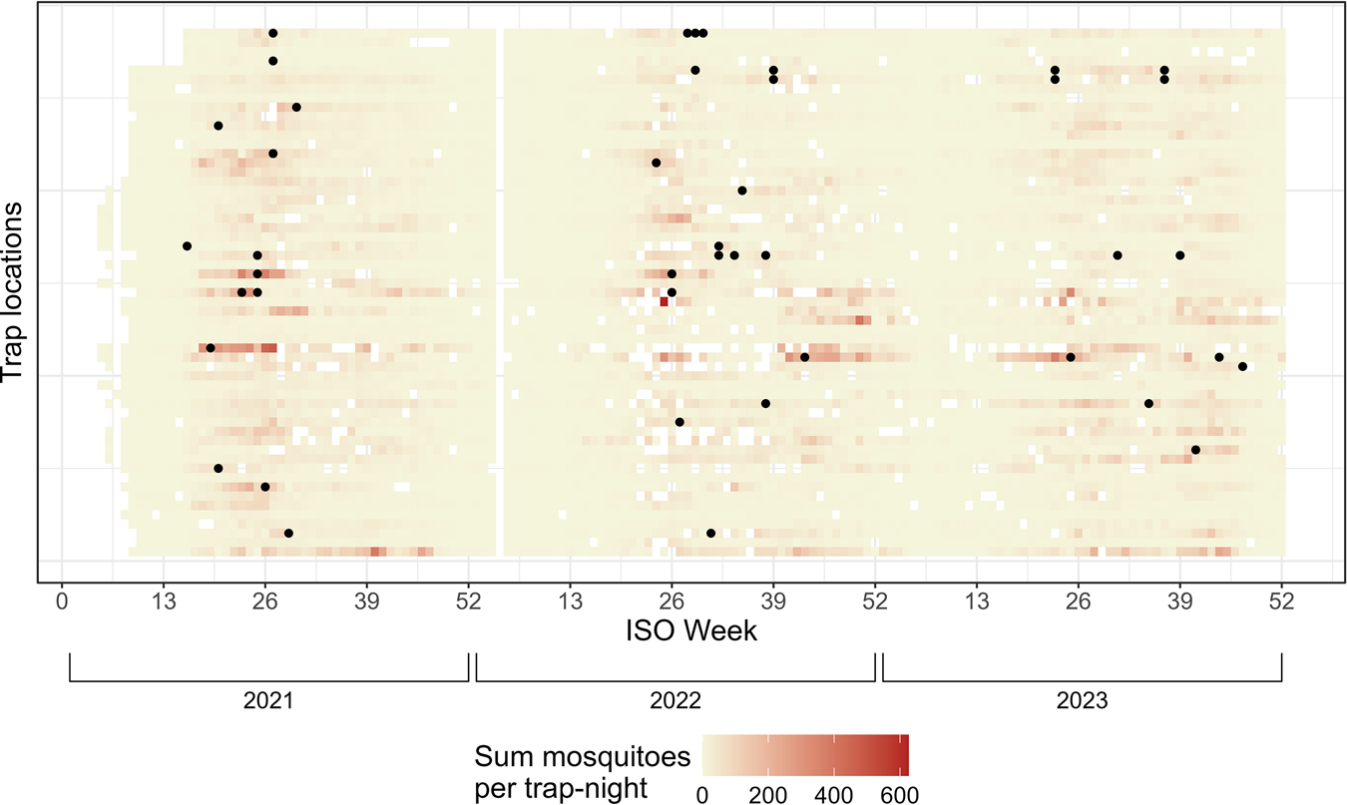
Heatmap of selected mosquito counts per trap and interventions per trap. Heatmap of the combined mosquito counts (*Cx. pipiens* and *Ae. albopictus*) per trap-night collected weekly during the three-year period from January 2021 through December 2023 (52 weeks per year). Darker colors indicate higher mosquito trap counts. Black circles represent weeks and respective sites in which an intervention against *Cx. pipiens* and/or *Ae. albopictus* took place.

The earliest intervention occurred during week 15 (mid-April) against *Cx. pipiens* and the latest intervention occurred during week 47 (late November) against both species. Mosquito counts of most trap locations never called for an intervention or only required a single intervention over the three-year period (Fig. 3). On three occasions, mosquito counts of a trap called for repeated interventions during the same week. Since the models considered the weekly binary occurrence of intervention, we considered these as a single intervention in the models.

### Interrupted time series models

Following variable selection, the AIC for the meta-regression models indicated that a smooth function of maximum temperature should be included in the individual, stratified models for *Cx. pipiens* and a smooth function of mean temperature should be included in the individual, stratified models for *Ae. albopictus*. However, the difference in AICs between the *Ae. albopictus* meta-regression models, which adjusted for mean temperature versus not including weather variables, were less than 1, thus we decided to use the more parsimonious models for this species. Individual model residuals no longer showed strong evidence of autocorrelation.

**Fig. 4:**
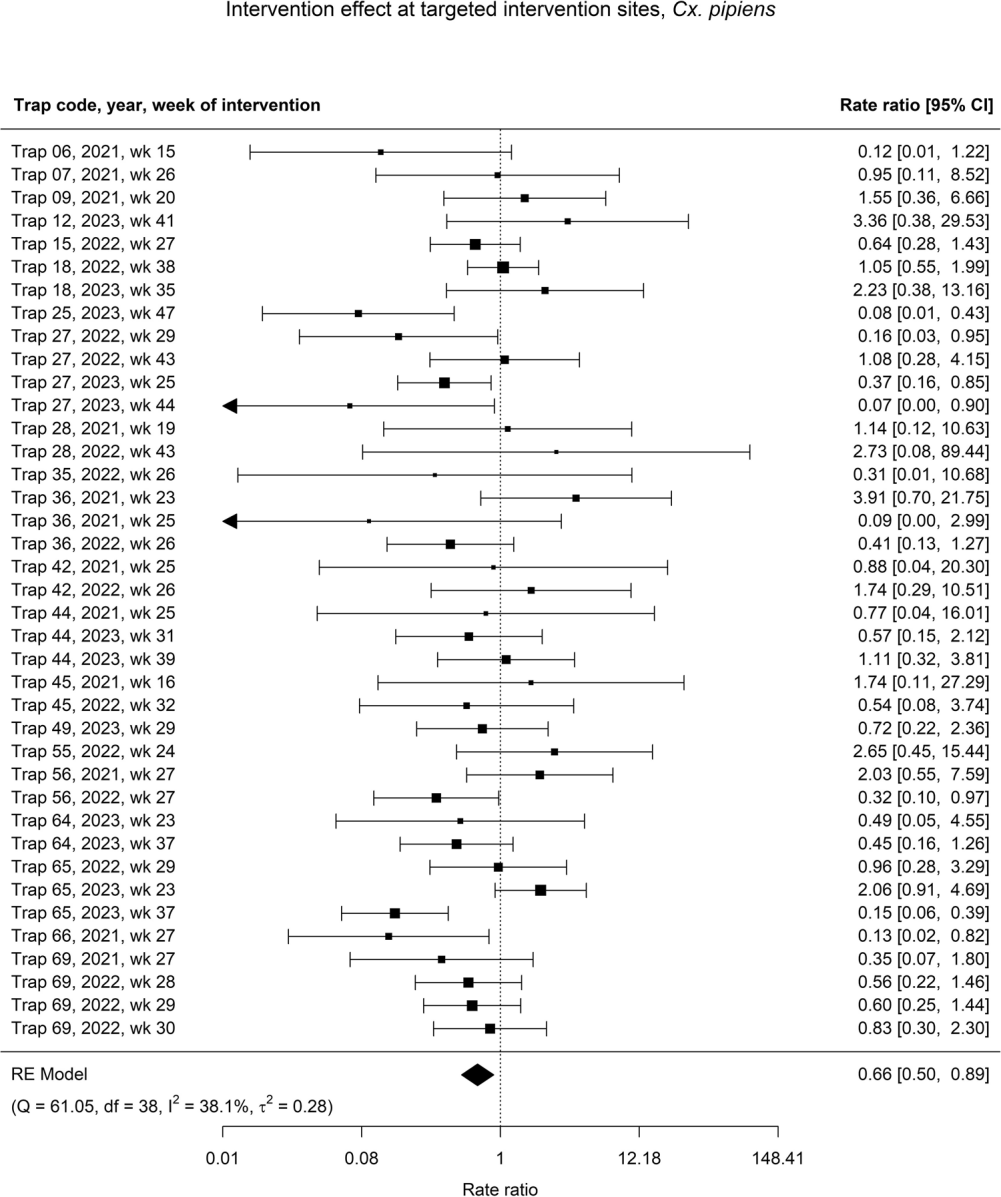
Forest plot of intervention effects on *Culex pipiens.* Forest plot of individual and pooled effect estimates of 39 IVM interventions on *Cx. pipiens* larvae and/or adults at intervention sites between January 2021 and December 2023. Unique interventions are identified by the trap code, year, and ISO week on the left of the figure, and rate ratios (RR) for the individual models’ effect estimates with their 95% confidence intervals (CI) are shown on the right. Error bars with arrows indicate estimates beyond the limits of the x-axis, which has been limited for visibility. Cochran’s Q test statistic and its associated degrees of freedom (df), as well as the τ^2^ and *I*^2^ values as indicators of heterogeneity in the meta-regression, are displayed below the plot.

Overall, deploying mosquito vector control interventions was associated with a significant 34% pooled reduction in *Cx. pipiens* adult counts (RR: 0.66, 95% CI: 0.50–0.89) compared to the pre-intervention time period (Fig. 4). We observed high heterogeneity between individual estimates, ranging from a 93% reduction (RR: 0.07, 95% CI: 0.00–0.90) at Trap 27 in week 44 of 2023 to a 291% increase (RR: 3.91, 95% CI: 0.70–21.75) at Trap 36 in week 23 of 2021 compared to the pre-intervention period (Fig. 4). At non-intervention sites less than 5 km from the intervention sites (hereafter: proximal sites), the post-intervention period was associated with a 23% pooled reduction in *Cx. pipiens* counts (RR: 0.77, 95% CI: 0.58–1.02) compared to the pre-intervention period (Supplementary Fig. 1). At non-intervention sites further than 5 km from the intervention sites (hereafter: non-proximal sites), the post-intervention period was associated with no change in *Cx. pipiens* counts (RR: 1.01, 95% CI: 0.88–1.15) compared to the pre-intervention period (Supplementary Fig. 2).

**Fig. 5:**
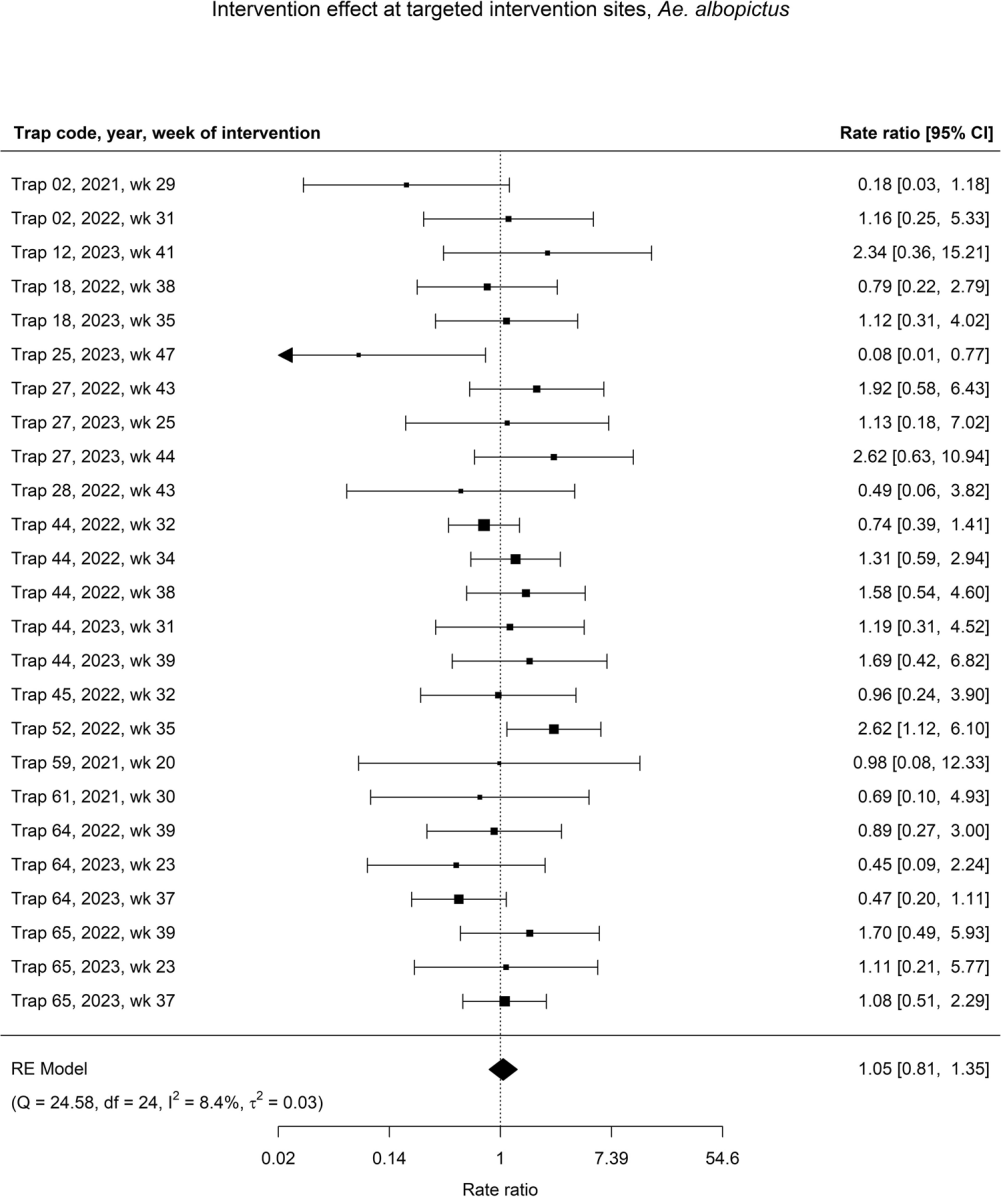
Forest plot of intervention effects on *Aedes albopictus.* Forest plot of the individual and pooled effect of 25 IVM interventions on *Ae. albopictus* larvae and/or adults at intervention sites between January 2021 and December 2023. Unique interventions are identified by the trap code, year, and ISO week on the left of the figure, and rate ratios (RR) for the individual models’ effect estimates with their 95% confidence intervals (CI) are shown on the right. Error bars with arrows indicate estimates beyond the limits of the x-axis, which has been limited for visibility. Cochran’s Q test statistic and its associated degrees of freedom (df), as well as the τ^2^ and *I*^2^ values as indicators of heterogeneity in the meta-regression, are displayed below the plot.

Following interventions against *Ae. albopictus*, we observed no change in adult counts (RR: 1.05, 95% CI: 0.81–1.35) compared to the pre-intervention period after pooling (Fig. 5). This pooled estimate did not come from significantly heterogeneous individual estimates. At the proximal and non-proximal sites of interventions targeting *Ae. albopictus*, we also did not observe significant differences in *Ae. albopictus* counts in the post-intervention period compared to the pre-intervention period (RR: 0.98, 95% CI: 0.84–1.13 and RR: 1.15, 95% CI: 0.94–1.41, respectively) (Supplementary Figs. 3 & 4).

The sensitivity analysis on stratifying by the life stage target of the intervention showed a nonsignificant 24% reduction in *Cx. pipiens* counts following interventions targeting only adult mosquitoes (RR: 0.76, 95% CI: 0.43–1.33). There were significant 38% and 52% reductions in *Cx. pipiens* counts following interventions targeting adults and larvae together (RR: 0.62, 95% CI: 0.41–0.93) and interventions targeting only larvae (RR: 0.48, 95% CI: 0.31–0.74) with a two-week lag, respectively. There remained no significant effect of any intervention type on *Ae. albopictus* counts (Supplementary Table 2). The sensitivity analysis on reducing the time period of the data subset from sixteen to eight weeks showed no pooled significant effect following interventions against either *Cx. pipiens* (RR: 1.00, 95% CI: 0.52–1.96) or *Ae. albopictus* (RR: 0.93, 95% CI: 0.70–1.25).

## Discussion

Our current study used mosquito surveillance data to assess the field effectiveness of an IVM program on entomological outcomes through a unique two-stage application of ITS analysis, in which individual intervention effect estimates were pooled to estimate the overall effect of the program. Our approach assessed a combination of control measures targeting both the adult and immature stages of the mosquito life cycle. Other studies evaluating the field effectiveness of mosquito vector control activities have typically focused on either adult or larval interventions^42–45^. Our analyses showed that the IVM program was successful in reducing *Cx. pipiens* populations, the most abundant mosquito vector found in the Attica Region for transmitting WNV, but showed limited success in controlling *Ae. albopictus* populations.

We observed *Cx. pipiens* in higher numbers for a longer period of the year and in a wider geographical range than *Ae. albopictus*, which is consistent with other studies of mosquito trap counts in the Mediterranean region where these species cohabitate^46,47^. The fluctuations in mosquito populations we observed from 2021 to 2023 could be attributed to yearly variations in weather conditions, particularly temperature, humidity, and precipitation. *Culex pipiens* and *Ae. albopictus* overwinters in the adult and egg stages, respectively^48^. Previous studies in the Attica Region, in the winter period of 2022–2023, revealed the presence of WNV overwintering in active *Cx. pipiens* captured in traps, in contrast with studies in other countries detecting WNV in hibernating mosquitoes^13^. Additionally, a previous study in the 2018–2019 winter demonstrated evidence of winter survival for *Ae. albopictus* females in human-made shelters in the Attica Region^29^. Notably, in the 2022–2023 winter, for the first time, adult *Ae. albopictus* remained active until January 2023, with December 2022 recording a significant trap count (714 adults) compared to previous years (e.g., 150 adults in December 2021)^49^. Monitoring mosquito populations during the winter months, as done in the Attica Region, is thus a crucial component of IVM for allowing early detection of the first generation in spring, enabling timely intervention of mosquito control activities.

For both species, we observed unequal bimodal peaks in trap counts in the weeks corresponding to early summer and mid-autumn. Both immature and adult survival of *Cx. pipiens*^50^ and *Ae. albopictus*^51^ are hindered at temperatures above 25 °C. The high summer temperatures and drought conditions in the Attica Region may have resulted in adult inactivity and consequently contributed to the mid-year (summer) decline in mosquito trap counts. Although we did not directly model weekly trap counts in our study, other studies in regions with Mediterranean climates have also observed bimodal peaks in mosquito trap counts^52,53^. By truncating the data to the weeks preceding and following each discrete intervention, we were able to reduce the variability associated with fluctuations in seasonal mosquito population dynamics and focus on the intervention effect itself. Although the overall pooled effect of interventions showed a significant reduction of *Cx. pipiens* trap counts, the individual effect estimates varied widely. Some discrete interventions were associated with increased *Cx. pipiens* counts compared to the pre-intervention period, though none of the estimates showing increased trap counts were statistically significant. One possible explanation for the observed increases could be the compensation effect phenomenon, wherein higher larval mortality counterintuitively results in higher adult mosquito trap counts^54–56^. Weather events such as heavy precipitation can also reduce the efficacy of insecticide treatments^57,58^. Only four intervention weeks in our data were associated with more than 15 mm of cumulative precipitation, so we could not make definitive conclusions about the role of rainfall in contributing to the heterogeneity we observed. Another possible explanation is the development of insecticide resistance, but this would require further investigation. Reducing the number of weeks in the sensitivity analysis likely exacerbated the effects of these specific circumstances, leading to the nonsignificant pooled result observed with eight-week subsets of the time series compared to sixteen-week subsets. The results from our analysis of proximal and non-proximal sites of the interventions targeting *Cx. pipiens* showed a trend towards null effect with increasing distance from the direct intervention, indicating a limited spatial carryover of the intervention effect, as has been observed previously in other settings^42^.

Larvae were not the sole target during interventions against *Ae. albopictus* alone in Attica Region, in contrast to IVM programs in other regions where this species has become established^44,59,60^. This could partially explain the reason why we did not observe a significant impact of the interventions on *Ae. albopictus* counts. Adult mosquito control through ultra-low volume (ULV) insecticide applications relies on the droplets making physical contact with the mosquitoes^61^. However, the resting behavior of *Ae. albopictus* significantly limits the effectiveness of this method^62,63^. Furthermore, *Ae. albopictus* tend to breed in small, cryptic water bodies often found in private areas, which would not be subjected to many of the interventions applied through the IVM program conducted on scale in public spaces^64^. Additionally, the higher trap counts and more widespread distribution of *Cx. pipiens* could also help explain the observed significant pooled reduction in *Cx. pipiens* counts but not *Ae. albopictus* counts.

Identifying characteristics of both successful and unsuccessful interventions can help program managers improve decision-making for the deployment of future interventions. The results of the sensitivity analysis on life stage target suggest that the effectiveness of interventions targeting *Cx. pipiens* was driven primarily by targeting larvae rather than adult mosquitoes. This can be further developed by using the model to identify high- and low-impact areas to better understand what worked or where improvements could be made, either to the timing or the type of intervention. Currently, the Attica Region follows the national action plan to manage WNV transmission risk, but there are only protocols in place to determine when interventions must be deployed, and nothing to determine which type of intervention should be used. Thus, there remains an opportunity to refine the program by integrating the knowledge learned through our current results into the action plan and developing systematic protocols for determining which interventions should be used within specific contexts. Furthermore, the cost-effectiveness of future interventions can be evaluated based on the observed effect size in this study using this methodological framework.

The management of invasive *Aedes* mosquito species is a complex process that requires implementation of established SOPs, raising public awareness, and offering comprehensive training resources^64,65^. Utilizing citizen science tools, such as engaging the public through the mobile phone application Mosquito Alert, can significantly enhance the effectiveness of monitoring and managing mosquito populations – an essential step for safeguarding public health, particularly with challenging species such as *Ae. albopictus*^66,67^. Future developments of the IVM program in the Attica Region will include the implementation of a tailored citizen science initiative to integrate the Mosquito Alert application in actively engaging the community in mosquito monitoring, fostering a collaborative effort to manage and control mosquito-related challenges in the Attica Region^68,69^. In addition, studies on the use of sterile insect technique (SIT) as intervention in Greece have shown promising results in managing *Ae. albopictus* populations^65,70,71^. Such methods could be further integrated into the invasive *Aedes* action plan, which already includes door-to-door inspections, larvicidal treatments in breeding sites that cannot be eliminated, and targeted adulticide spraying.

The next step in evaluating the effectiveness of Attica Region’s IVM program is to consider its impact on epidemiological outcomes such as disease transmission. A study in California (USA) demonstrated the effectiveness of aerial ULV treatments at reducing *Culex* populations as well as WNV infection rates in collected mosquitoes^72^, which could correlate to an overall reduction in human WNV incidence as mosquito infections have been associated with transmission to humans^73,74^. Similarly, the implementation of an intensified IVM program was associated with a decrease in *Ae. aegypti* and *Ae. albopictus* abundance indices in Sri Lanka, where *Aedes* spp. abundance is much higher than in Europe^75^, and associations have been observed between mosquito and human infections for dengue virus in endemic regions^76^. Tangentially, there have been no human WNV cases in Attica Region since 2021, when three cases were reported in two areas of the region, corresponding to incidence rates of 1.8 and 5.9 per 100,000^77^. This suggests that the successful control of *Cx. pipiens* through the IVM program may have contributed to this reduction, but follow-up studies are necessary to quantify this impact and integrate this information into IVM program protocols to prioritize interventions based on epidemiological outcomes.

Our current findings indicate that the IVM program in the Attica Region, Greece, has been effective in reducing mosquito populations, particularly *Cx. pipiens*. The implementation of monitoring protocols for all mosquito species on a permanent basis, with a focus on species of public health significance, may serve as a reference in entomological surveillance activities for a target area. Using systematically collected entomological data, decision-making can be supported, helping to ensure and sustain the successful implementation of the IVM program to contain mosquito vector populations of public health concern. This comprehensive approach can provide a reliable model for countries or regions considering the development or improvement of existing VBD prevention and control practices.

## Methods

### Study area

**Fig. 6:**
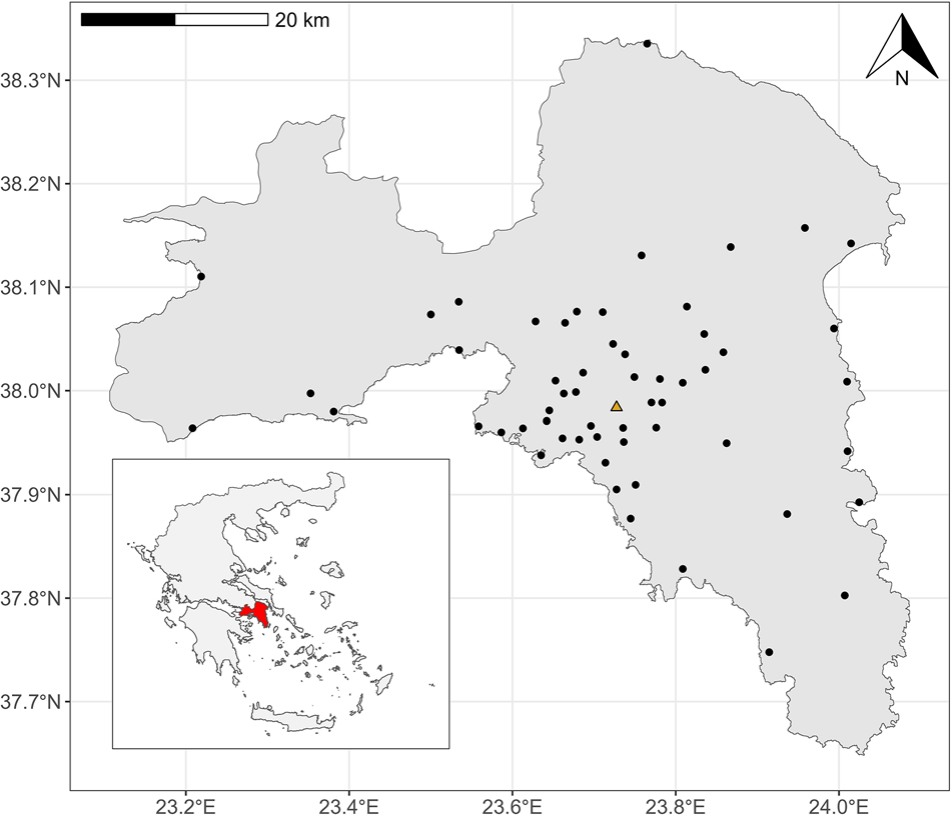
Map of the study area. Map of the study area. Black circles represent the 57 BG-Sentinel trap locations, and the yellow triangle represents the centroid coordinates of Athens. The inset map shows the location of the Attica Region, highlighted in red, within Greece. Shapefile boundaries were sourced from GADM, version 4.1^97^.

The study area consisted of Attica Region in Greece (Fig. 6). Attica Region is a peninsula in the Aegean Sea within the Mediterranean climate zone, and is characterized by hot, dry summers and cool, wet winters^78^. Despite being mostly mountainous and arid, the Attica Region is the location of the capital city, Athens, making the region densely populated and urbanized. Furthermore, some protected wetland areas exist, such as Schinias National Park in the northeast.

### Data collection and management

Our team at the Laboratory of Insects and Parasites of Medical Importance of the BPI conducted weekly mosquito sampling at the 57 stationary trap locations in predominantly urban and peri-urban locations throughout Attica Region from 2021 to 2023 (Fig. 6). Through funding from the “Research project for the entomological surveillance of mosquitoes in Attica Region (Athens, Greece)”, we expanded mosquito population monitoring to ensure comprehensive geographical coverage across Attica Region. The entomological surveillance of adult mosquitoes was performed on a weekly basis using a network of continuously operating BG-Sentinel traps baited with both CO_2_ and the BG lure (BioGents, Regensburg, Germany), in selected, fixed sampling sites^79^. At the conclusion of each sampling event, we collected the trap net containing captured mosquitoes and placed it on ice to preserve the specimens for further analysis. The mosquitoes were transported to BPI for careful examination, counting, and sexing under a stereoscope to ensure accurate morphological identification^80–82^.

In addition, we established an oviposition network consisting of 110 ovitraps. We placed two ovitraps at 55 of the 57 BG-Sentinel trap locations, at least 50 meters apart from each other and at least 50 meters away from the BG-Sentinel traps. Weekly sampling of ovitraps was conducted according to standard operating procedures (SOPs)^64,83^. We transferred wooden substrates from the field to the laboratory, and *Aedes* eggs were counted and recorded. This surveillance strategy enabled simultaneous monitoring of adult mosquitoes via BG-Sentinel traps and eggs from *Ae. albopictus* invasive mosquito species via ovitraps throughout the study period.

For the statistical analyses presented here, we only considered the total number of adult female *Cx. pipiens* and *Ae. albopictus*. Sampling events in which the trap experienced a malfunction or other problem that invalidated the count (e.g., ants in the trap) were excluded from analyses.

### Mosquito control interventions

Most European countries implement mosquito control programs at national, regional, and local levels. In most cases, private Pest Control Operators (PCOs) selected through public tenders are responsible for implementing interventions and appropriate tools and approaches for efficient control. Most PCOs are certified by the International Organization of Standardization (ISO), which defines general standards to ensure the quality, safety, and efficiency of products, services, and systems, as well as traceability^84^. In the Attica Region, vector control has relied almost exclusively on larviciding campaigns executed by private contractors (PCOs) selected through competitive tenders issued by the Regional Authorities. Adulticiding was strategically reserved as an additional intervention, primarily when larvicidal interventions had minimal effect on the vector population or for verified epidemic amplification and imported cases of *Aedes*‑borne diseases such as dengue, chikungunya, and Zika^84^.

Ιn our current study, interventions where biocides were used (Supplementary Table 1) followed EU regulations, particularly Regulation (EU) No 528/2012 (also known as the Biocidal Products Regulation). The list of registered biocides in the EU, for both mosquito larvae and adult control, is publicly available on the European Chemicals Agency (ECHA) and the Greek Ministry of Rural Development and Food websites^85,86^. The PCOs are mandated to use only the registered biocidal products (larvicidal or adulticidal), strictly in accordance with their marketing authorization and current labeling. Therefore, Regional authorities certify proper use, as any use not specified in the marketing authorization and labeling is expressly prohibited and subject to criminal and administrative penalties under applicable legislation. For the purpose of this study, we recorded only whether the intervention targeted larvae or adults, not the specific product name or active ingredient. The outcome of this approach is more generic and can also be applied in the future since the list of registered products in the EU is constantly modified (mainly for commercial products and much less for active ingredients).

While IVM programs emphasize evidence-based decision-making for mosquito vector control interventions, specific, standardized criteria for initiating such actions are not globally established. The World Health Organization suggests a decision-making process to optimize resource use in mosquito vector control, aiming to increase the efficiency, cost-effectiveness, and sustainability without providing a threshold for action initiation^87^. Therefore, in the absence of a threshold, our process involved evaluating whether the number of individuals recorded in a particular trap exceeded the overall weekly average captures from all active traps for that week, in combination with the average number of captures recorded by that specific trap over the previous five to seven weeks, proposing a replicable framework in standardized decision-making criteria for IVM programs. The timeframe was defined as five weeks if the trap was continuously operational and seven weeks if the trap had been inactive for at least one week during that period. Where interventions were deemed necessary based on mosquito counts, these were enacted within a 50- to 200-m radius of the trap’s location. The exact range and type of intervention were determined by land use characteristics and environmental factors, such as urban density and vegetation cover. This proximity ensured direct spatial correlation between the intervention zones and monitoring locations. Employing this methodology, we tried to ensure that decisions were data-driven, considering both temporal trends and the specific context of each trap’s placement.

### Data management for model development

We transformed the recorded surveillance observations to a weekly time series by taking the week number of the trap collection date of each collection event, following the International Organization for Standardization (ISO) definition of week numbers, where week 1 is the week that contains the first Thursday of January. To complete the time series for each species, we imputed missing counts by predicting values from an autoregressive model of order one with the week of the time series (1-156) stratified by trap location as the only random effect^88^. We only imputed missing counts if fewer than 10% of the observations for a given trap location were missing. The number of trap-nights for each collection event was calculated as the number of days between the trap installation date and the trap collection date. We standardized mosquito counts by dividing the count by the number of trap-nights and used the count per trap-night as the outcome of our analyses.

We matched the dates and type of mosquito control spatially by trap location and temporally by week and year to the mosquito count time series and gave a binary code, i.e., an intervention week at the respective trap(s) was coded as 1, and weeks with no intervention were coded as 0. Interventions were also species-specific. Thus, analyses matched the intervention target to the species being modeled.

We retrieved meteorological variables from the ERA5-Land reanalysis grid at 0.1° resolution^89^. The hourly mean temperature, maximum temperature, minimum temperature, and total precipitation values from January 2020 through December 2023 were extracted from the closest valid grid points to each trap’s coordinates and summarized into daily values. We aggregated daily values weekly to match the temporal scale of the mosquito collection data by taking the mean, maximum, minimum, and sum of daily values, respectively.

### Model development

We developed separate models for each species *Cx. pipiens* and *Ae. albopictus* as an interrupted time series (ITS) model for the outcome mosquito count per trap-night. Interrupted time series models allowed us to estimate the immediate impact of an intervention and typically require a time series with an intervention at a defined point in time^90^. Therefore, we conducted a two-stage analysis in which each unique week when mosquito vector control was deployed was treated as a discrete intervention.

In the first stage, we identified each unique week in the full time series in which an intervention of any type was enacted and created data subsets of the site-specific time series around each of these weeks, beginning seven weeks before and ending eight weeks after the intervention. In total, there were 39 weeks with interventions targeting *Cx. pipiens* and 25 weeks with interventions targeting *Ae. albopictus*. Fifteen interventions appeared in the data subsets for both species because they were enacted in response to high trap counts of both species concurrently. One intervention, which targeted both species, occurred during week 47 of 2023; thus, the post-intervention period for this intervention was truncated to five weeks by the extent of the data. To estimate the effect of the interventions, we introduced a binary indicator variable that took the values of 0 for the weeks before the intervention and 1 from the week of the intervention until the end of the data subset. For each data subset, we fitted generalized additive models (GAM) following a negative binomial distribution for the outcome mosquito count per trap-night, and the restricted estimated maximum likelihood (REML) method was used for smoothing parameter estimation^91^. Due to negative binomial models requiring integers as the outcome, we rounded the count per trap-night to the nearest integer, with any values between 0 and 1 rounded to 1. The models considered smooth functions of temperature (mean/maximum/minimum) and total precipitation, as well as linear coefficients of these variables as covariates. We checked model residuals to make sure there was no evidence of autocorrelation. This procedure resulted in 39 discrete models for *Cx. pipiens* and 25 discrete models for *Ae. albopictus*, from which we extracted the individual effect estimates for the intervention indicator variable.

In the second stage, we pooled the resulting intervention effect estimates in an inverse variance-weighted random effects meta-regression model to produce an overall effect estimate of intervention on mosquito counts. The covariate combination in the stratified models, which resulted in the lowest Akaike information criterion (AIC) for the meta-regression on the effect estimate of intervention, was retained^92^.

In order to compare trap locations that did and did not require mosquito control interventions in a given week, we repeated the two-stage analysis for sites less than 5 km from the intervention site but where no intervention occurred, as well as non-intervention sites further than 5 km from the intervention sites. When more than one non-intervention site was present, we included a random effect for the trap in the model. Additional models were not fitted if no sites were within 5 km of a given intervention.

Finally, we conducted two sensitivity analyses. The first evaluated the species-specific meta-regression estimates by pooling intervention effect estimates that were derived from interventions against adult mosquitoes only, larvae only, or adults and larvae together to determine whether intervention type affected the estimated effectiveness of the intervention. When examining interventions targeting only larvae, the individual models were re-fitted with a two-week lag introduced for the intervention indicator variable to account for a delay in observing the effects of larval control against adult mosquito counts. The second evaluated the time period for creating the data subsets by reducing from sixteen to eight weeks of data in each individual model.

We conducted all analyses and visualizations in R version 4.3.1^93^ using the packages “mgcv”^91^, “metaphor”^94^, “sf”^95^, and “ggplot2”^96^.

### Inclusion & Ethics

This study was conducted jointly between researchers at Heidelberg University, Germany, and the Benaki Phytopathological Institute, Greece. This collaboration is acknowledged by the shared roles for PCS and GB as well as for AM and AB.

### Reporting summary

Further information on research design is available in the Nature Portfolio Reporting Summary linked to this article.

## Supplementary information

Supplementary information

Reporting summary

## Data Availability

The entomological data that support the findings of this study are available in the public repository (10.6084/m9.figshare.28497380). Meteorological data from the ERA5-Land reanalysis for 2020-2023 can be found at: https://cds.climate.copernicus.eu/datasets/reanalysis-era5-land?tab=overview.

## Code availability

The codes to produce the models and figures in this paper can be found at 10.6084/m9.figshare.28497380.
