# Supplementary material for: Three-year assessment of integrated vector management in Attica, Greece: results from surveillance and control activities
Source: Commun Biol. 2025 Nov 6;8:1533. doi: 10.1038/s42003-025-08825-y (PMC12592716; doi:10.1038/s42003-025-08825-y)
Supplement: Supplementary file 1 — Supplementary information [file 42003_2025_8825_MOESM1_ESM.pdf]

Supplementary information

**Supplementary Table 1** Operational details of each intervention. The products used varied by intervention, but all biocides used comply with EU regulations, particularly Regulation (EU) No 528/2012. The list of registered biocides for both mosquito larvae and adult control is available on the ECHA website (accessible in all EU languages): https://echa.europa.eu/information-on-chemicals/biocidal-active-substances.

| Year, Week | Trap code | Species targeted | Intervention method | Life stage target |
|---|---|---|---|---|
| 2021, 15 | 06 | *Culex pipiens* | Eliminate breeding sites | Larvae |
| 2021, 16 | 45 | *Culex pipiens* | Eliminate breeding sites | Larvae |
| 2021, 19 | 28 | *Culex pipiens* | Silicone liquid | Larvae |
| 2021, 20 | 59 | *Aedes albopictus* | Eliminate breeding sites, Biocides against adults and larvae | Both |
| 2021, 20 | 09 | *Culex pipiens* | Eliminate breeding sites, Biocides against adults | Both |
| 2021, 23 | 36 | *Culex pipiens* | Eliminate breeding sites, Biocides against larvae | Both |
| 2021, 25 | 36 | *Culex pipiens* | Eliminate breeding sites, Biocides against larvae | Larvae |
| 2021, 25 | 44 | *Culex pipiens* | Biocides against larvae | Larvae |
| 2021, 25 | 42 | *Culex pipiens* | Eliminate breeding sites, Biocides against larvae | Larvae |
| 2021, 26 | 07 | *Culex pipiens* | Eliminate breeding sites, Biocides against larvae | Larvae |
| 2021, 27 | 56 | *Culex pipiens* | Door to door campaign | Larvae |
| 2021, 27 | 66 | *Culex pipiens* | Biocides against larvae, Door to door campaign | Larvae |
| 2021, 27 | 69 | *Culex pipiens* | Biocides against adults and larvae | Both |
| 2021, 29 | 02 | *Aedes albopictus* | Biocides against adults and larvae | Both |
| 2021, 30 | 61 | *Aedes albopictus* | Biocides against adults | Adults |
| 2022, 24 | 55* | *Culex pipiens* | Biocides against adults and larvae | Both |
| 2022, 26 | 42 | *Culex pipiens* | Biocides against adults | Adults |
| 2022, 26 | 35 | *Culex pipiens* | Biocides against adults | Adults |
| 2022, 26 | 36 | *Culex pipiens* | Eliminate breeding sites | Larvae |
| 2022, 27 | 56 | *Culex pipiens* | Biocides against adults and larvae | Both |
| 2022, 27 | 15 | *Culex pipiens* | Eliminate breeding sites | Larvae |
| 2022, 28 | 69 | *Culex pipiens* | Biocides against adults and larvae | Both |
| 2022, 29 | 69 | *Culex pipiens* | Biocides against adults and larvae | Both |
| 2022, 29 | 27 | *Culex pipiens* | Biocides against adults and larvae, Door to door campaign | Both |
| 2022, 29 | 65 | *Culex pipiens* | Biocides against adults | Adults |

| | | | | |
|---|---|---|---|---|
| 2022, 30 | 69 | *Culex pipiens* | Biocides against adults and larvae | Both |
| 2022, 31 | 02 | *Aedes albopictus* | Biocides against adults and larvae | Both |
| 2022, 32 | 45 | Both species | Biocides against adults | Adults |
| 2022, 32 | 44* | *Aedes albopictus* | Biocides against adults and larvae | Both |
| 2022, 34 | 44 | *Aedes albopictus* | Biocides against adults | Adults |
| 2022, 35 | 52 | *Aedes albopictus* | Biocides against adults | Adults |
| 2022, 38 | 44* | *Aedes albopictus* | Biocides against adults, Door to door campaign | Both |
| 2022, 38 | 18 | Both species | Biocides against adults | Adults |
| 2022, 39 | 65 | *Aedes albopictus* | Biocides against adults | Adults |
| 2022, 39 | 64 | *Aedes albopictus* | Biocides against adults | Adults |
| 2022, 43 | 27 | Both species | Biocides against adults | Adults |
| 2022, 43 | 28 | Both species | Biocides against adults | Adults |
| 2023, 23 | 64 | Both species | Biocides against larvae | Larvae |
| 2023, 23 | 65 | Both species | Biocides against adults | Adults |
| 2023, 25 | 27 | Both species | Eliminate breeding sites | Larvae |
| 2023, 29 | 49 | *Culex pipiens* | Eliminate breeding sites | Larvae |
| 2023, 31 | 44 | Both species | Biocides against adults | Adults |
| 2023, 35 | 18 | Both species | Eliminate breeding sites | Both |
| 2023, 37 | 64 | Both species | Biocides against adults | Adults |
| 2023, 37 | 65 | Both species | Biocides against adults | Adults |
| 2023, 39 | 44 | Both species | Biocides against larvae | Larvae |
| 2023, 41 | 12 | Both species | Eliminate breeding sites | Larvae |
| 2023, 44 | 27 | Both species | Eliminate breeding sites | Larvae |
| 2023, 47 | 25 | Both species | Eliminate breeding sites | Larvae |

*These interventions were repeated twice in the same week. For modelling purposes, they were considered as a single intervention week.

**Supplementary Table 2** Pooled effect estimates following the sensitivity analysis of stratifying the meta-regression analysis by life stage target of the intervention.

| | *Culex pipiens* | | *Aedes albopictus* | |
|---|---|---|---|---|
| | **N*** | **RR (95% CI)*** | **N*** | **RR (95% CI)*** |
| All interventions | 39 | 0.66 (0.50-0.89) | 25 | 1.05 (0.81-1.35) |
| Larvae only** | 18 | 0.48 (0.31-0.74) | 7 | 1.13 (0.36-3.50) |
| Adults and larvae together | 10 | 0.62 (0.41-0.93) | 5 | 0.84 (0.51-1.36) |
| Adult mosquitoes only | 11 | 0.76 (0.43-1.33) | 13 | 1.12 (0.80-1.57) |

*N: number of interventions; RR: rate ratio; CI: confidence interval)

**The individual models were re-fitted to introduce a two-week lag to the intervention indicator variable.

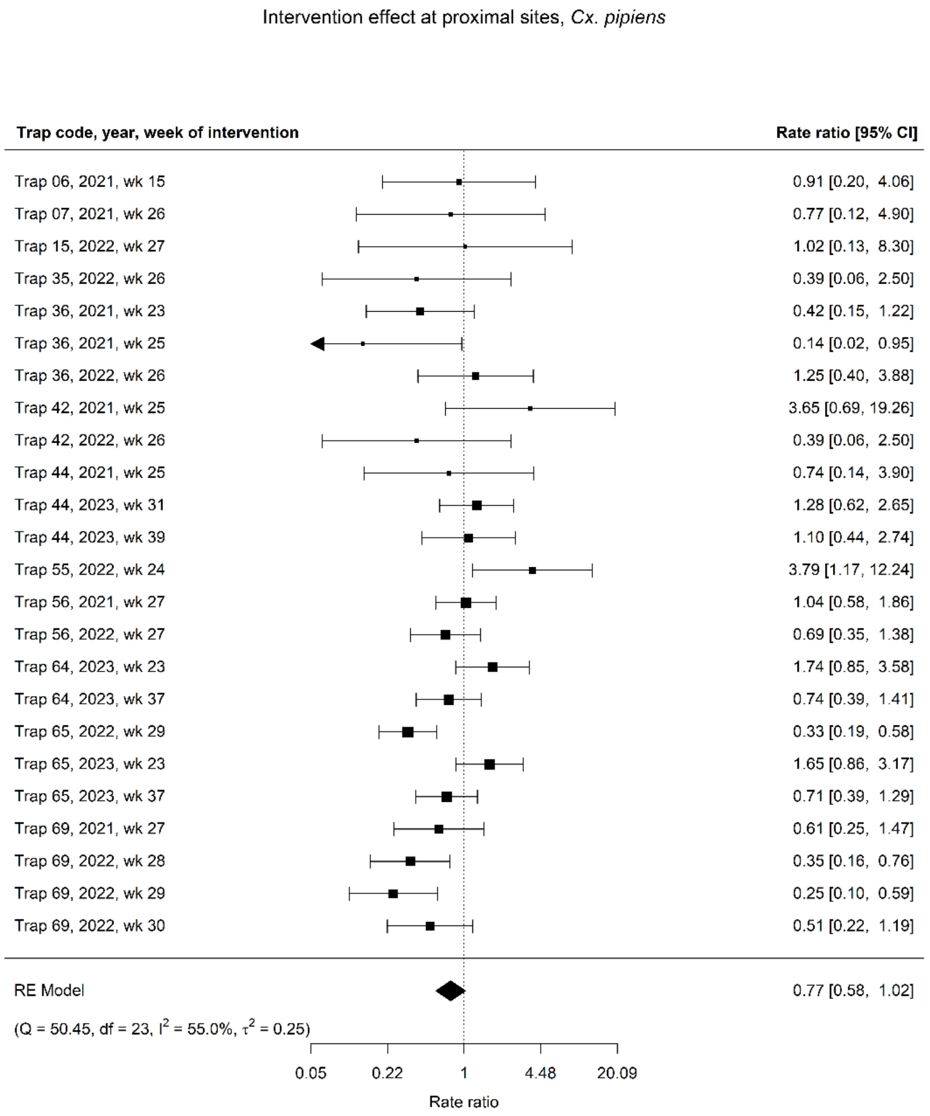


**Supplementary Figure 1** Forest plot of individual and pooled effect estimates of 39 IVM interventions on *Cx. pipiens* counts at proximal sites, which were non-intervention sites less than 5 km from intervention sites, between January 2021 and December 2023. Unique interventions are identified by the trap code, year, and ISO week on the left of the figure and rate ratios (RR) for the individual models' effect estimates with their 95% confidence intervals (CI) are shown on the right. Error bars with arrows indicate estimates beyond the limits of the x-axis, which has been limited for visibility. The Cochran's Q test statistic and its associated degrees of freedom (df) as well as the $\tau^2$ and $I^2$ values as indicators of heterogeneity in the meta-regression are displayed below the plot.

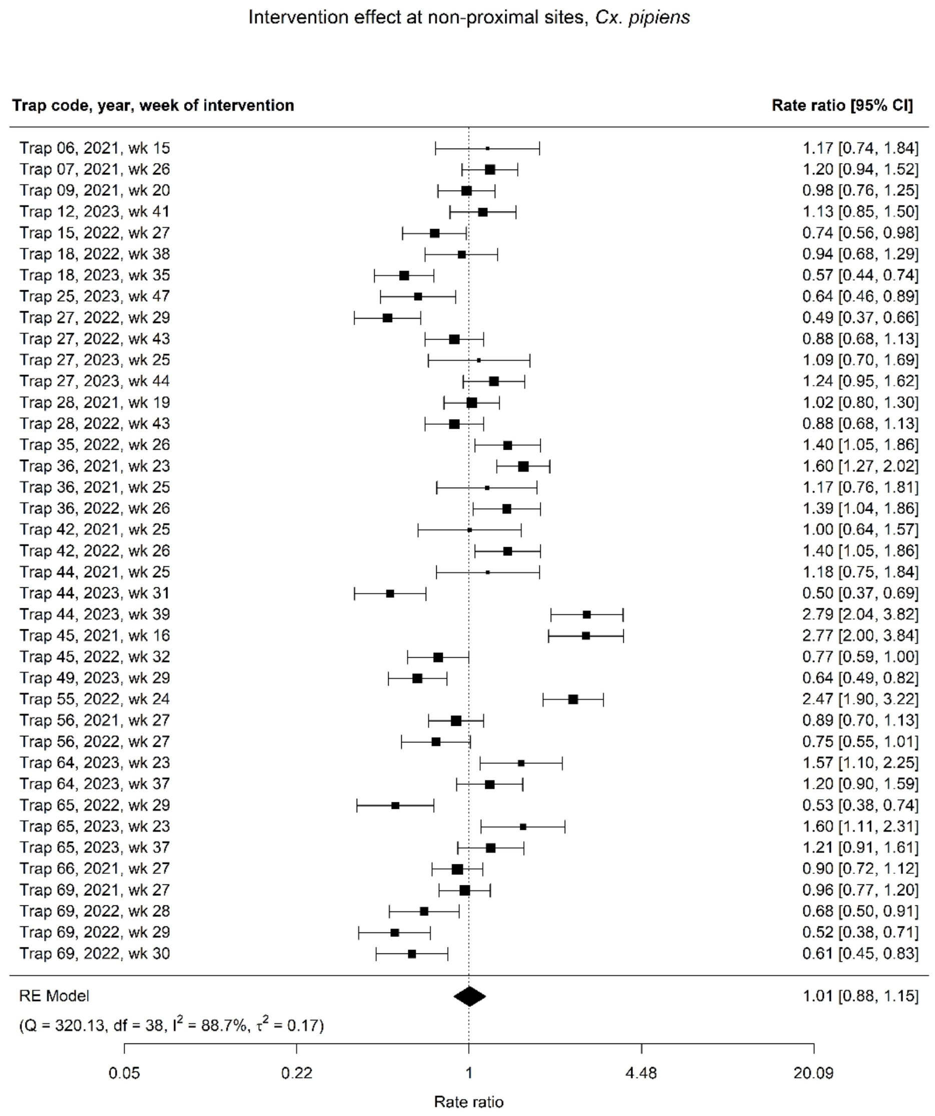


**Supplementary Figure 2** Forest plot of individual and pooled effect estimates of 39 IVM interventions on *Cx. pipiens* counts at non-proximal sites, which were non-intervention sites more than 5 km from intervention sites, between January 2021 and December 2023. Unique interventions are identified by the trap code, year, and ISO week on the left of the figure and rate ratios (RR) for the individual models' effect estimates with their 95% confidence intervals (CI) are shown on the right. The Cochran's Q test statistic and its associated degrees of freedom (df) as well as the $\tau^2$ and $I^2$ values as indicators of heterogeneity in the meta-regression are displayed below the plot.

Intervention effect at proximal sites, *Ae. albopictus*

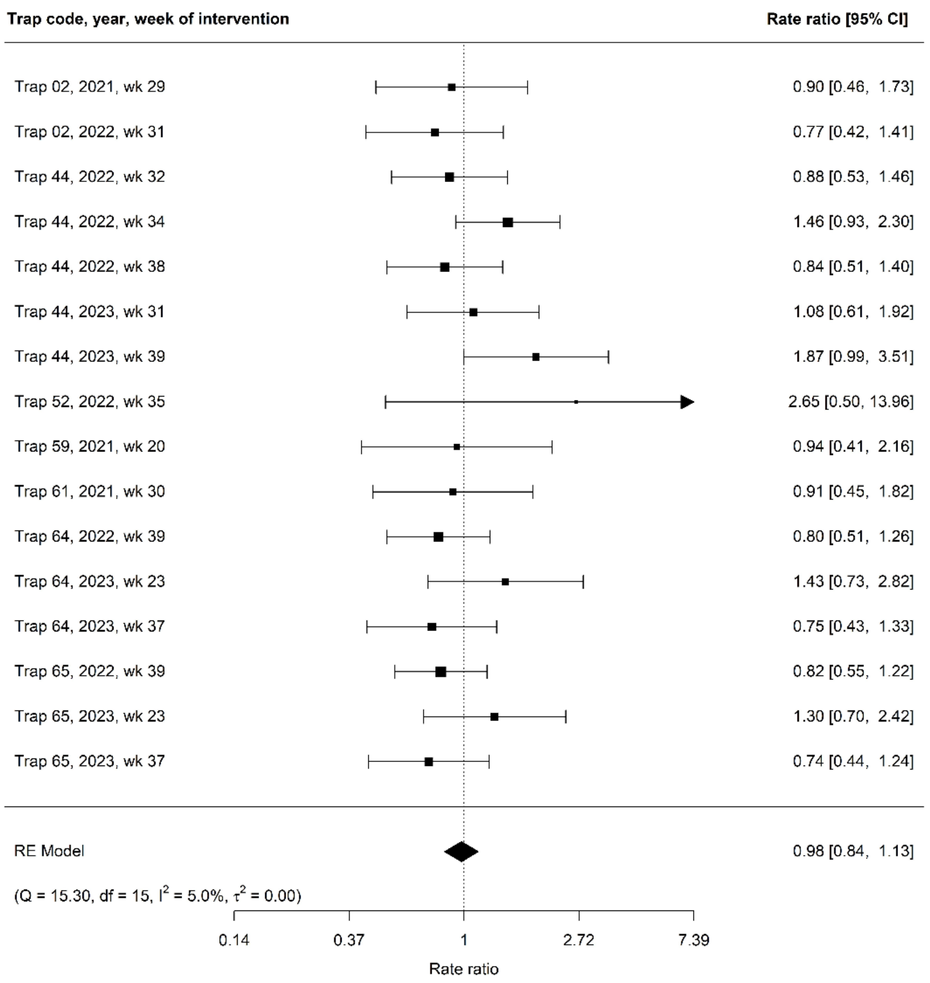


**Supplementary Figure 3** Forest plot of individual and pooled effect estimates of 25 IVM interventions on *Ae. albopictus* counts at proximal sites, which were non-intervention sites less than 5 km from intervention sites, between January 2021 and December 2023. Unique interventions are identified by the trap code, year, and ISO week on the left of the figure and rate ratios (RR) for the individual models' effect estimates with their 95% confidence intervals (CI) are shown on the right. Error bars with arrows indicate estimates beyond the limits of the x-axis, which has been limited for visibility. The Cochran's Q test statistic and its associated degrees of freedom (df) as well as the $\tau^2$ and $I^2$ values as indicators of heterogeneity in the meta-regression are displayed below the plot.

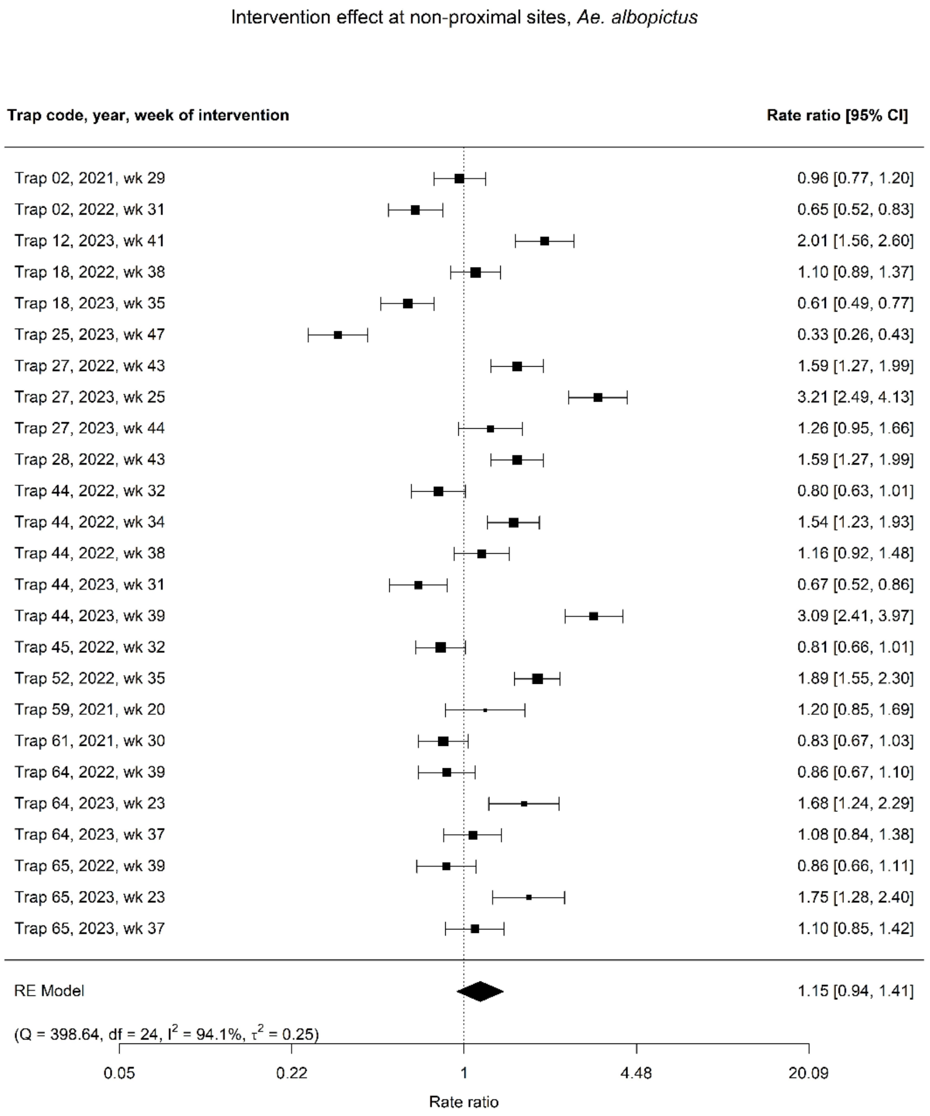


**Supplementary Figure 4** Forest plot of individual and pooled effect estimates of 25 IVM interventions on *Ae. albopictus* counts at non-proximal sites, which were non-intervention sites more than 5 km from intervention sites, between January 2021 and December 2023. Unique interventions are identified by the trap code, year, and ISO week on the left of the figure and rate ratios (RR) for the individual models' effect estimates with their 95% confidence intervals (CI) are shown on the right. The Cochran's Q test statistic and its associated degrees of freedom (df) as well as the $\tau^2$ and $I^2$ values as indicators of heterogeneity in the meta-regression are displayed below the plot.
