# Supplementary material for: Three-year assessment of integrated vector management in Attica, Greece: results from surveillance and control activities
Source: Commun Biol. 2025 Nov 6;8:1533. doi: 10.1038/s42003-025-08825-y (PMC12592716; doi:10.1038/s42003-025-08825-y)
Supplement: Supplementary file 2 — Reporting summary [file 42003_2025_8825_MOESM2_ESM.pdf]

Corresponding author(s): Aditi Bunker, Antonios Michaelakis

Last updated by author(s): Aug 22, 2025

Reporting Summary

Nature Portfolio wishes to improve the reproducibility of the work that we publish. This form provides structure for consistency and transparency in reporting. For further information on Nature Portfolio policies, see our Editorial Policies and the Editorial Policy Checklist.

Statistics

For all statistical analyses, confirm that the following items are present in the figure legend, table legend, main text, or Methods section.

n/a	Confirmed	
☐	☒	The exact sample size (n) for each experimental group/condition, given as a discrete number and unit of measurement
☐	☒	A statement on whether measurements were taken from distinct samples or whether the same sample was measured repeatedly
☒	☐	The statistical test(s) used AND whether they are one- or two-sided<br>*Only common tests should be described solely by name; describe more complex techniques in the Methods section.*
☐	☒	A description of all covariates tested
☒	☐	A description of any assumptions or corrections, such as tests of normality and adjustment for multiple comparisons
☐	☒	A full description of the statistical parameters including central tendency (e.g. means) or other basic estimates (e.g. regression coefficient) AND variation (e.g. standard deviation) or associated estimates of uncertainty (e.g. confidence intervals)
☒	☐	For null hypothesis testing, the test statistic (e.g. F, t, r) with confidence intervals, effect sizes, degrees of freedom and P value noted<br>*Give P values as exact values whenever suitable.*
☒	☐	For Bayesian analysis, information on the choice of priors and Markov chain Monte Carlo settings
☒	☐	For hierarchical and complex designs, identification of the appropriate level for tests and full reporting of outcomes
☐	☒	Estimates of effect sizes (e.g. Cohen's d, Pearson's r), indicating how they were calculated

Our web collection on statistics for biologists contains articles on many of the points above.

Software and code

Policy information about availability of computer code

Data collection	No software was used.
Data analysis	We conducted all analyses and visualizations in R version 4.3.1 using the packages “mgcv”, “metafor”, “sf”, and “ggplot2”. The codes to produce the models and figures in this paper can be found at https://doi.org/10.6084/m9.figshare.28497380.

For manuscripts utilizing custom algorithms or software that are central to the research but not yet described in published literature, software must be made available to editors and reviewers. We strongly encourage code deposition in a community repository (e.g. GitHub). See the Nature Portfolio guidelines for submitting code & software for further information.

Data

Policy information about availability of data

All manuscripts must include a data availability statement. This statement should provide the following information, where applicable:

- Accession codes, unique identifiers, or web links for publicly available datasets
- A description of any restrictions on data availability
- For clinical datasets or third party data, please ensure that the statement adheres to our policy

The entomological data that support the findings of this study are available in the public repository (https://doi.org/10.6084/m9.figshare.28497380). Meteorological data from the ERA5-Land reanalysis for 2020-2023 can be found at: https://cds.climate.copernicus.eu/datasets/reanalysis-era5-land?tab=overview.

Research involving human participants, their data, or biological material

Policy information about studies with human participants or human data. See also policy information about sex, gender (identity/presentation), and sexual orientation and race, ethnicity and racism.

Reporting on sex and gender	NA
Reporting on race, ethnicity, or other socially relevant groupings	NA
Population characteristics	NA
Recruitment	NA
Ethics oversight	NA

Note that full information on the approval of the study protocol must also be provided in the manuscript.

Field-specific reporting

Please select the one below that is the best fit for your research. If you are not sure, read the appropriate sections before making your selection.

☐ Life sciences ☐ Behavioural & social sciences ☒ Ecological, evolutionary & environmental sciences

For a reference copy of the document with all sections, see nature.com/documents/nr-reporting-summary-flat.pdf

Ecological, evolutionary & environmental sciences study design

All studies must disclose on these points even when the disclosure is negative.

Study description	We used a two-stage interrupted time series approach to estimate the individual and pooled effects of mosquito control interventions on mosquito counts estimated through routine surveillance.
Research sample	The research sample consisted of field-collected counts of mosquitoes through a routine mosquito surveillance program in the region of Attica, Greece, as well as logged mosquito control interventions. The primary species collected were Culex pipiens and Aedes albopictus, which are both vectors of human arboviruses.
Sampling strategy	The selection of trap placement sites was conducted using the geostatistical method of stratified random sampling, with land use serving as the stratification criterion.
Data collection	The 57 traps were generally sampled on a weekly basis throughout the collection period, except for occasional instances where accessibility issues prevented collection. Collected adult specimens were morphologically identified using standard taxonomic keys
Timing and spatial scale	The first samples were collected on 5 Feb 2021 and the last samples were collected on 29 Dec 2023. The 57 trap locations were generally sampled weekly during the collection period, apart from sporadic instances of accessibility issues which prevented collection. The traps were stationary points which were consistent throughout the study period.
Data exclusions	Sampling events in which the trap experienced a malfunction or other problem which invalidated the count (e.g., ants in the trap) were excluded from analyses.
Reproducibility	Mosquito collection and identification followed standard operating procedures, and codes for conducting the analyses are stored in an open-access repository.
Randomization	The data were not randomized because the application of mosquito control was conducted in response to elevated public health risk of vector-borne disease transmission, thus it was not ethical to designate certain surveillance sites as control and withhold treatment.
Blinding	Blinding was not relevant to this study as the intervention of mosquito control was not randomly allocated.

Did the study involve field work? ☒ Yes ☐ No

Field work, collection and transport

Field conditions	Field conditions in the Attica region were characterized by a mix of urban and peri-urban environments, presenting varied microhabitats conducive to mosquito breeding and necessitating adaptive sampling strategies.

Location	The study area was the Attica Region in Greece, a peninsula extending into the Aegean Sea within the Mediterranean climate zone. Attica is characterized by hot, dry summers and cool, wet winters. Additionally, the region includes several protected wetland areas, such as Schinias National Park located in the northeast.
Access & import/export	Through funding from the "Research project for the entomological surveillance of mosquitoes in Attica Region (Athens, Greece)", we expanded mosquito population monitoring to ensure comprehensive geographical coverage across Attica.
Disturbance	Sampling events in which the trap experienced a malfunction or other problem which invalidated the count (e.g., ants in the trap) were excluded from analyses.

Reporting for specific materials, systems and methods

We require information from authors about some types of materials, experimental systems and methods used in many studies. Here, indicate whether each material, system or method listed is relevant to your study. If you are not sure if a list item applies to your research, read the appropriate section before selecting a response.

Materials & experimental systems

n/a	Involved in the study
☒	☐ Antibodies
☒	☐ Eukaryotic cell lines
☒	☐ Palaeontology and archaeology
☒	☐ Animals and other organisms
☒	☐ Clinical data
☒	☐ Dual use research of concern
☒	☐ Plants

Methods

n/a	Involved in the study
☒	☐ ChIP-seq
☒	☐ Flow cytometry
☒	☐ MRI-based neuroimaging

Plants

Seed stocks	*Report on the source of all seed stocks or other plant material used. If applicable, state the seed stock centre and catalogue number. If plant specimens were collected from the field, describe the collection location, date and sampling procedures.*
Novel plant genotypes	*Describe the methods by which all novel plant genotypes were produced. This includes those generated by transgenic approaches, gene editing, chemical/radiation-based mutagenesis and hybridization. For transgenic lines, describe the transformation method, the number of independent lines analyzed and the generation upon which experiments were performed. For gene-edited lines, describe the editor used, the endogenous sequence targeted for editing, the targeting guide RNA sequence (if applicable) and how the editor was applied.*
Authentication	*Describe any authentication procedures for each seed stock used or novel genotype generated. Describe any experiments used to assess the effect of a mutation and, where applicable, how potential secondary effects (e.g. second site T-DNA insertions, mosiacism, off-target gene editing) were examined.*
